# Septic Pulmonary Embolism With Deep Vein Thrombosis and Bilateral Pleural Effusion

**DOI:** 10.7759/cureus.29437

**Published:** 2022-09-21

**Authors:** Mrinmayee V Mayekar, Ulhas S Jadhav, Babaji Ghewade, Pankaj B Wagh, Gaurang M Aurangabadkar

**Affiliations:** 1 Respiratory Medicine, Jawaharlal Nehru Medical College, Datta Meghe Institute of Medical Sciences, Wardha, IND; 2 Respiratory Medicine, Datta Meghe Medical College, Datta Meghe Institute of Medical Sciences, Wardha, IND

**Keywords:** virchow’s triad, pulmonary ct angiography, d-dimer, septic pulmonary emboli, deep vein thrombosis (dvt)

## Abstract

Septic emboli induce two insults - firstly, the infectious insult, which causes inflammation and increases the potential for abscess formation, and secondly, the early embolic/ischemic insult brought on by arterial blockage and infarction. Pulmonary embolism is the second leading cause of cardiovascular disease-associated death, right after cardiovascular events. The sequelae of venous thromboembolism include post-thrombotic syndrome and chronic thromboembolic pulmonary hypertension (CTEPH). The pathophysiological characteristics of inflammation, hypercoagulability, and endothelial damage are the three components of Virchow's triad, which are mirrored by the risk factors for both diseases. The screening of patients for whom venous thromboembolism can be ruled out with a positive plasma D-dimer test result is made easier by clinical probability evaluation. To confirm the diagnosis, compression ultrasonography that displays deep vein thrombosis or a chest CT that reveals pulmonary embolism have been frequently employed. We report a case of a young male who presented with sudden onset pain and swelling in the left lower limb and chest following an intramuscular injection which further resulted in pulmonary thromboembolism.

## Introduction

Pulmonary thromboembolism is a condition in which a blood clot primarily from the deep venous system of the lower limbs gets lodged in the pulmonary arteries leading to sudden onset of chest pain and breathlessness. Septic emboli result in two insults-the early embolic/ischemic insult due to vascular occlusion, which may lead to infarction, and the infectious insult that leads to inflammation and possible abscess formation. Pulmonary embolism is a medical emergency and requires quick diagnosis and intervention as it can have a fatal outcome [1].

A rare form of pulmonary embolism termed septic pulmonary embolism (SPE) emerges when pathogen-containing emboli travel to the pulmonary arteries and cause pulmonary embolism and localized lung abscesses. More catheter-related SPE reports have been published due to the expanding usage of intravascular devices and catheters [1]. Pulmonary thromboembolism can have acute as well as chronic presentations depending on the cause. After cardiovascular events, pulmonary embolism ranks third amongst other causes of cardiovascular disease-associated death [2]. Chronic thromboembolic pulmonary hypertension (CTEPH) and post-thrombotic syndrome are sequelae of venous thromboembolism [2]. Septic emboli have an extensive range of presentations from asymptomatic to severe with high mortality. Deep venous thrombosis and pulmonary embolism are both included under the overarching framework of venous thromboembolism [3]. A combination of inheritable and acquired risk factors may also trigger thromboembolic events [3].

## Case presentation

A 27-year-old male patient was brought to the emergency department with primary complaints of sudden-onset stabbing type of pain in the left lower limb, preceded by swelling. The next day, the patient experienced worsening breathlessness which progressed to Modified Medical Research Council (mMRC) grade IV. The patient also had high-grade fever with chills and persistent dry cough for 2-3 days.

On examination, his pulse rate was 138 beats/min, with oxygen saturation (SpO_2_%) of 80% in room air with a temperature of 101 degrees Fahrenheit (F). The left leg of the patient was swollen, and tenderness was present over the left calf region. A difference of 5 cm was observed between the left and the right calf diameters. On chest auscultation, bilateral decreased breath sounds and basal crepitations were found. The abdominal, neurological, and cardiovascular examinations were within normal limits. All routine blood investigations were found to be within normal ranges.

**Figure 1:**
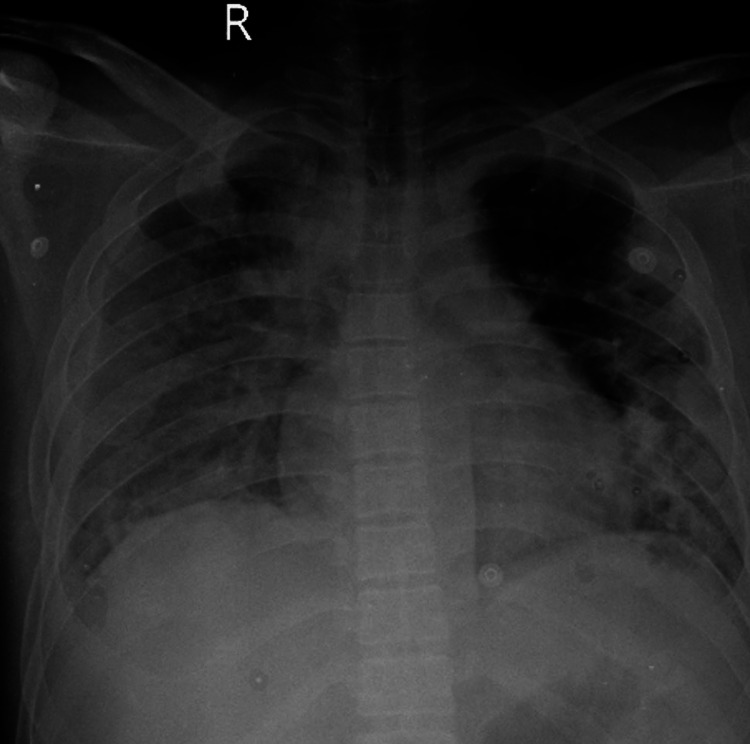
Chest x-ray posteroanterior (PA) view on admission suggestive of bilateral pulmonary infiltrates with right-sided pleural effusion and haziness in the left lower zone

Sputum was sent for acid-fast bacilli (AFB) and Gene Xpert which was also negative for mycobacterium tuberculosis. For further evaluation, a bilateral lower limb color Doppler was done, which was suggestive of deep vein thrombosis (DVT) involving the left popliteal and calf veins with mild to moderate subcutaneous soft tissue edema over the left lower limb with cellulitis. A chest x-ray posteroanterior (PA) view was done upon admission which showed bilateral lung infiltrates with right-sided moderate pleural effusion (Figure 1).

**Figure 2:**
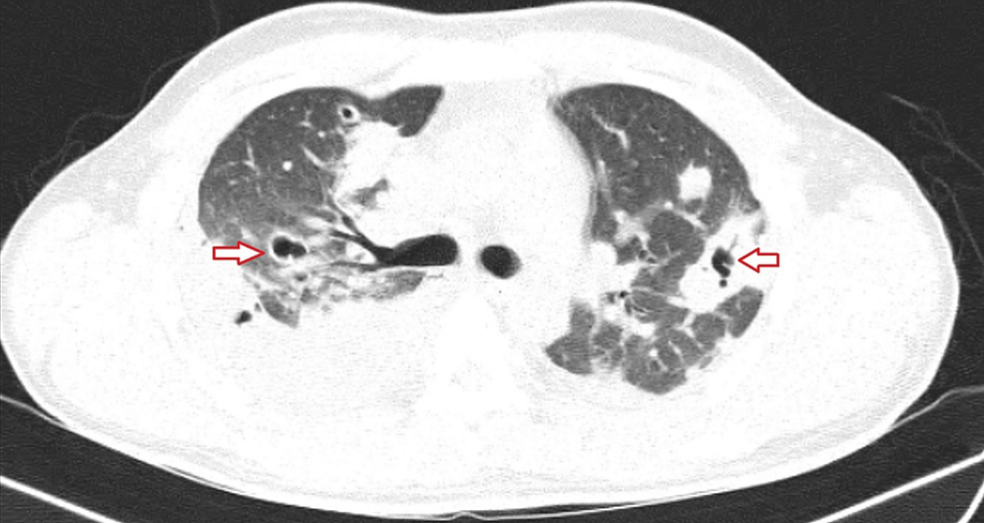
High-resolution computerized tomography (HRCT) of the thorax (lung window) showing multiple cavitary lesions in more in the right middle lobe and the left upper lobe (red arrows)

**Figure 3:**
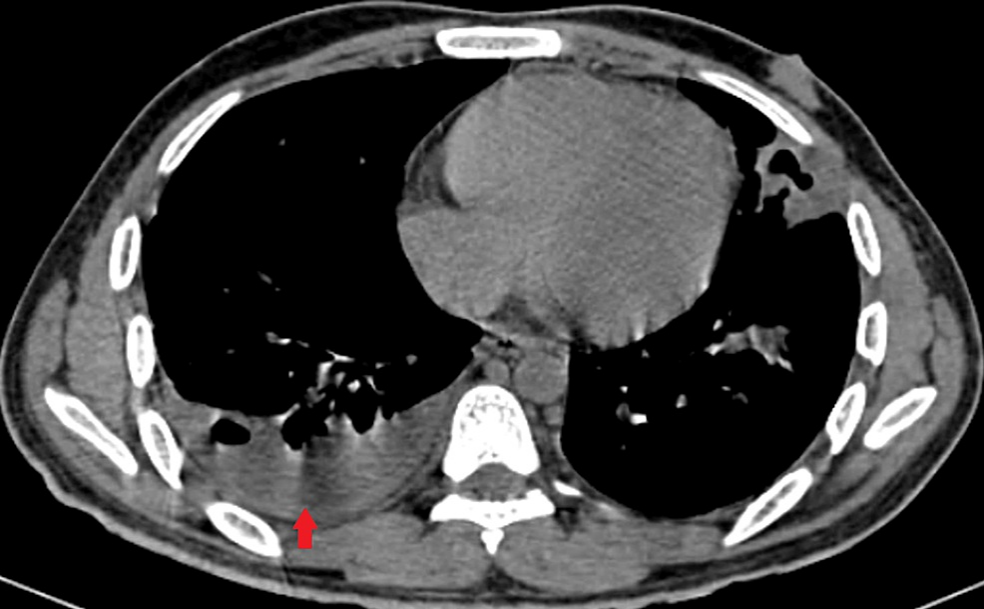
High-resolution CT thorax (mediastinal window) showing right-sided moderate pleural effusion (red arrow)

High-resolution computerized tomography (HRCT) of thorax was done for the patient which was suggestive of bilateral moderate pleural effusion with multiple cavitary lesions primarily in the left upper and right middle lobe with wedge-shaped consolidation (Figures 2, 3).

 

**Figure 4:**
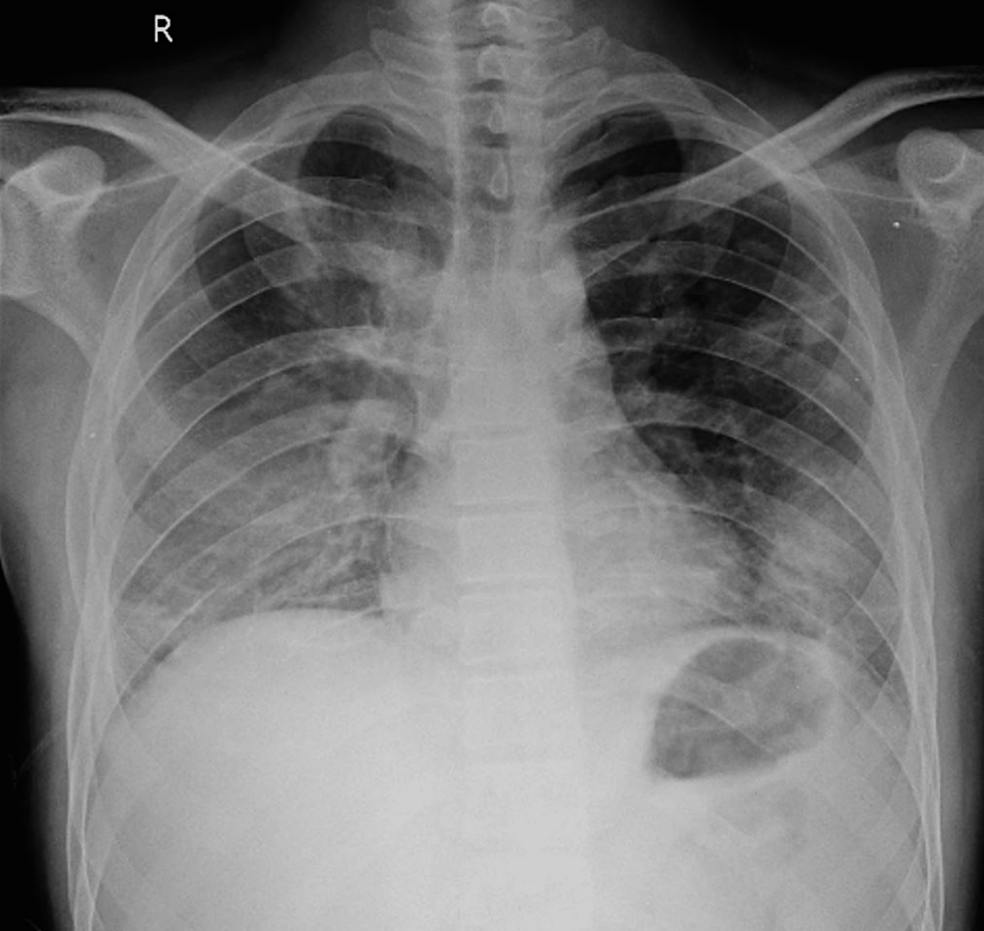
Chest x-ray posteroanterior (PA) view, post-thoracic pigtail catheter insertion on the right side, showing improvement in the right-sided pleural effusion

Later, ultrasound-guided pigtail catheter insertion was done on the right side. Post-pigtail insertion chest x-ray was done which showed improvement in the right-sided pleural effusion (Figure 4).

Approximately 1,500 mL of hemorrhagic fluid was drained post-pigtail insertion. The pleural fluid culture was done which identified the growth of Methicillin-sensitive staphylococcus aureus (MSSA). According to the culture sensitivity report, the patient was started on Piperacillin - Tazobactam and low molecular weight heparin (LMWH), and other symptomatic treatment to which he responded well and was discharged after 15 days on oral anticoagulants, antibiotics, and supportive therapy and was asked to follow-up after 15 days.

## Discussion

SPE is a very infrequent clinical condition caused by implantation in the pulmonary vasculature with reinfection and embolization of lytic pathogens mixed with fibrin from the site of infection to the venous circulation. Infarctions and micro-abscesses are driven by the embolization of microorganisms containing thrombi into the pulmonary vascular beds, which results in SPE [2]. The most common etiological factors leading to the development of SPE are summarised in Table 1 [3].

**Table 1:** Common etiological factors leading to septic pulmonary embolism (SPE)

Infectious Endocarditis
Skin and Soft tissue infections
Cardiac pacemakers
Intravenous drug abuse
Dental abscesses
Septic thrombophlebitis
Puerperal sepsis

A typical radiographic finding on a CT scan of the chest shows bilateral solitary nodules with varying degrees of cavitation; the size of the nodules may vary due to recurring embolic showers. The feeding vessel sign, which is a unique vessel that leads directly to the nodule, and subpleural wedge-shaped opacities are two new findings that might be found and are considered pathognomonic for SPE [3-5]. Pleural effusion or empyema may occasionally complicate SPE. The radiological indicators of SPE can be confused with those of several other illnesses, notably pulmonary vasculitis (granulomatosis with polyangiitis), extra-thoracic metastases (often squamous cell carcinoma), and pulmonary thromboembolism.

The duration of the disease and its clinical manifestations are crucial clues in pinpointing its etiologic cause. An infectious etiology is seen when a clinical picture with a cavity evolves quickly (within 12 weeks), but a chronic or indolent course (lasting more than 12 weeks) is more likely to imply malignancy or vasculitis. The key to treatment is determining the actual reason and executing the appropriate course of action. DVT should always be looked for when a patient has respiratory symptoms in conjunction with musculoskeletal pain, especially if staphylococcal infection is suspected. With conflicting data about the relevance of an isolated S. aureus bacteremia and the risk of endocarditis, an echocardiogram should indeed be planned to be carried out in this clinical scenario [4-6]. Pulmonary thromboembolism is less common in young adults without any pre-disposing causes; thus, the case becomes a little challenging, and detailed history gains importance. The various complications and differential diagnoses of cavitary pulmonary lesions are summarized in Table 2 [5].

**Table 2:** Complications and differential diagnosis of cavitary pulmonary lesions

Complications of Cavitary lung lesions	Differential diagnosis of Cavitary lung lesions
Empyema	Cavitary pulmonary metastases
Pneumothorax	Necrotizing pneumonia
Bronchopleural fistula	Granulomatosis with polyangiitis
Hemoptysis	Pulmonary tuberculosis

## Conclusions

The presence of septic thrombophlebitis may be found adjacent to the primary anatomical focus of infection and may also lead to SPE. Due to the presence of cavitary pulmonary lesions, the radiological signs of pulmonary thromboembolism can mimic Wegener's granulomatosis, and therefore proper history taking and understanding of the course of disease progression is of the utmost importance. In this patient who presented with an acute history of dyspnea, after detailed clinical examination, it became evident that a local site infection could have led to septic emboli resulting in pulmonary thromboembolism which was managed with broad-spectrum antimicrobial therapy and anticoagulants without the need for active surgical intervention. Therefore, it is essential to have a high index of suspicion for SPE in patients with primary infective focus anywhere in the body, who show rapidly worsening dyspnea and oxygen saturation. Early initiation of oxygen support, appropriate antimicrobial drugs, and anti-coagulant therapy can improve the prognosis of the patient and can contribute to a reduction in mortality.
